# Young children with psychotic symptoms and risk for suicidal thoughts and behaviors: a research note

**DOI:** 10.1186/s13104-018-3680-3

**Published:** 2018-08-10

**Authors:** Keneisha Sinclair-McBride, Nicholas Morelli, Sahil Tembulkar, Kelsey Graber, Joseph Gonzalez-Heydrich, Eugene J. D’Angelo

**Affiliations:** 1 Department of Psychiatry, Boston Children’s Hospital, 300 Longwood Avenue, Boston, MA 02115 USA; 2 Harvard Medical School, 25 Shattuck Street, Boston, MA 02115 USA; 3 Department of Pediatrics, Stanford University School of Medicine, 300 Pasteur Drive, Stanford, CA 94305 USA

**Keywords:** Suicide, Suicidal behaviors, Children, Psychosis, Clinical high risk

## Abstract

**Objective:**

Suicidal thoughts and behaviors (STBs) are prevalent among youth with psychotic disorders (PD) relative to the general population. Recent research now suggests that STBs may present during the prodromal phase of the disease, or the clinical high risk (CHR) state. While this knowledge is important for the development of suicide prevention strategies in adolescent and adult populations, it remains unclear whether risk for suicide extends to children with or at risk for psychosis. The current study is an extension of previous work assessing STBs in youth across the psychosis continuum. We examine STBs in 37 CHR and PD children ages 7–13 years old, and further explore the prodromal symptom correlates of STB severity among CHR children.

**Results:**

CHR and PD children endorsed STBs with a frequency and severity similar to what is observed in older CHR and PD populations. A number of children had never previously vocalized their suicidal plans or intent. Among CHR children, *Social Anhedonia* and *Odd Behavior or Appearance* were significantly correlated with STB severity. These findings underscore the importance of screening for STBs even in young children presenting with psychotic symptoms.

## Introduction

Suicide is a well-established risk among individuals with psychotic disorders, accounting for approximately 5–10% of mortality [1, 2]. Recent efforts toward early identification and prevention reveal that suicidal behaviors frequently occur before the onset of acute psychotic symptoms during the prodromal phase, or clinical high risk (CHR) state—a premorbid period of subthreshold positive psychotic symptoms, brief psychotic symptoms, or cognitive decline combined with a family history of psychosis. As is seen in psychotic disorders, suicide risk during the CHR state is high relative to the general population. Approximately 66% of CHR individuals report suicidal ideation, while 18% will make a suicide attempt [3]. Previous work has sought to identify specific psychotic-like prodromal symptoms that are associated with suicidal thoughts and behaviors, with mixed findings; some studies suggest that positive symptoms are correlated with suicide risk [4], while others implicate negative symptoms [5]. To date, no research has investigated correlates of suicidal behaviors among CHR children.

Roughly one-third of psychotic disorders develop prior to adulthood [6]. These cases are typically categorized as either early onset psychosis (EOP; onset between 13 and 17 years of age) or very early onset psychosis (VEOP; onset before 13 years of age). While the utility of this distinction remains contentious [7], there is evidence that VEOP, even compared to EOP, is associated with a range of negative outcomes, including poorer academic achievement, greater unemployment, and longer hospitalizations [8, 9]. A small number of papers find elevated rates of suicidal behaviors in VEOP comparable to those seen in older psychotic individuals [10, 11]. However, these studies excluded children who did not meet strict criteria for schizophrenia, limiting our understanding of suicide risk in children experiencing psychoses more broadly (e.g., schizoaffective disorder, schizophreniform disorder, etc.).

The current study is an exploratory extension of a previous project assessing suicidal behaviors in youth with and at risk for psychosis [12, 13]. Here, we focus on the youngest subsample of participants (children ages 7–13 years) at CHR and with a diagnosed psychotic disorder (PD). Specifically, we investigate the rates of suicidal ideation, planning, attempts, and self-reported predictions of future attempts (herein referred to as *suicidal thoughts and behaviors*; *STBs*), in an effort to guide future research on this understudied population. Additionally, we further explore the CHR group to see whether, in this preliminary study, prodromal symptoms correlate with STBs. No study to our knowledge has attempted to explore suicidal thoughts, behaviors, reporting tendencies, and STB symptom correlates in an age range of CHR and PD children this young.

## Main text

### Methods

Thirty-seven (37) help-seeking children (n = 21 CHR and n = 16 PD) ages 7–13 years old were included in the study. Participants were recruited as part of a larger study on neuroplasticity and psychosis [14] from referring clinicians in a large university-affiliated children’s hospital. Recruitment targeted children reporting psychotic symptoms; referrals were not made on the basis of suicidal thoughts or behaviors. All participants were evaluated using the schedule for affective disorders and schizophrenia for school-age children—present and lifetime version (KSADS-PL) [15]. Those who met criteria for a psychotic disorder not in the context of substance use or other medical condition were included in the PD group, while those with subthreshold psychotic symptoms were administered the structured interview for prodromal syndromes (SIPS) [16]. CHR status was determined on the basis of meeting criteria for at least one of three SIPS psychosis risk syndromes. To minimize study visit length, those who were diagnosed with a psychotic disorder via the KSADS-PL were not administered the SIPS. Exclusion criteria included intellectual disability, history of neurobiological disorders, and previous traumatic brain injury. Informed consent was obtained for all parents. Child participants provided assent following Frost et al.’s guidelines on the ethical inclusion of children with psychosis in research [17]. The study was approved by the Institutional Review Board.

The KSADS-PL [15] was used to assess current and past psychiatric disorders for all participants. The SIPS [16] was used to assess subthreshold psychotic symptoms and determine CHR status, which was defined by three possible prodromal syndromes: (1) attenuated positive risk syndrome (APS): a score of 3–5 on the scale of prodromal symptoms (SOPS); (2) genetic risk and deterioration syndrome (GRDS): a first-degree relative with psychosis coupled with functional deterioration; or (3) Brief intermittent psychotic syndrome (BIPS): the presence of psychotic symptoms that are brief and do not meet diagnostic threshold for a psychotic disorder. The SIPS assesses four categories of prodromal symptoms: positive, negative, disorganized, and general. STBs were evaluated using the Suicide Behaviors Questionnaire—Revised (SBQ-R) [18], a brief, four-item self-report measure that assesses (1) previous (lifetime) suicide attempts and plans, (2) frequency of suicidal ideation in the past 12 months, (3) past (lifetime) vocalization of suicide intent or plans, and (4) future likelihood of suicide attempt. Participants receive an SBQ-R total severity score from 3 to 22, with scores above 7 representing elevated suicide risk with 87% sensitivity and 93% specificity in clinical populations [18].

### Results

Sociodemographic information is presented in Table 1. Eleven (11) CHR children (52.4%) and 13 PD children (81.3%) met criteria for at least one comorbid psychiatric disorder, including 6 mood disorders in the CHR group (28.6%) and 2 in the PD group (12.5%). The psychotic disorders for which PD participants met criteria were psychotic disorder NOS (not otherwise specified; n = 8), schizoaffective disorder (n = 5), schizophrenia (n = 1), schizophreniform disorder (n = 1), and bipolar I disorder with psychotic features (n = 1).

**Table 1:** Socio-demographic information

	CHR (n = 21)	PD (n = 16)	Difference between groups
Gender (female/male/trans)	12/8/1	4/12/0	*χ*^2^ = 4.41*
Age: mean (SD)	10.6 (1.8)	10.1 (1.6)	*t*_35_ = .87
Household income			N/A
$0–$39,999	5 (23.8%)	5 (31.3%)	
$40,000–$99,999	6 (28.6%)	5 (31.3%)	
> $100,000	7 (33.3%)	3 (18.8%)	
No response	3 (14.3%)	2 (12.5%)	
Race/ethnicity (%)			N/A
White	16 (76.2%)	12 (75.0%)	
Black	1 (4.8%)	0 (0%)	
Asian American	1 (4.8%)	0 (0%)	
Native American	0 (0%)	1 (9.5%)	
Latino	2 (9.5%)	1 (9.5%)	
Multiracial	0 (0%)	2 (12.5%)	
No response	1 (4.8%)	0 (0%)	

Sociodemographics by study classification are listed above. There were no group differences in age. Males made up a significantly larger portion of the PD group than the CHR group. Cells were too small for formal group comparisons by household income or race/ethnicity

*CHR* clinical high risk, *PD* psychotic disorder, *SD* standard deviation

* p < .05

#### Suicidal thoughts and behaviors

STBs were evident in both study groups. Of the 21 CHR children, 5 (23.8%, 95% CI [5.6%, 42.0%]) scored at or above the SBQ-R total score cutoff. Ten (47.6%, 95% CI [26.2% 69.0%]) reported past year suicidal ideation, and four (19.0%, 95% CI [2.2%, 35.8%]) reported having previously attempted suicide. Five (23.8%, 95% CI [5.6%, 42.0%]) CHR children reported a non-zero likelihood of a future suicide attempt. Of the 16 PD children, 8 (50.0%, 95% CI [25.5%, 74.5%]) scored at or above the cutoff. Eleven (68.6%, 95% CI [45.9%, 91.3%]) reported past year ideation, with 4 (25.0%, 95% CI [3.8%, 46.2%]) reporting a previous suicide attempt. Four (25.0%, 95% CI [3.8%, 46.2%]) PD children reported a non-zero likelihood of a future suicide attempt.

Responses on SBQ-R item 4 (“Have you ever told someone that you were going to commit suicide, or that you might do it”) indicated that a number of participants who endorsed suicidal thoughts or behaviors had never previously reported it. Of the 6 children who endorsed making a suicide plan at one point in their lifetime, 5 (2 CHR and 3 PD) had never vocalized this to someone else. Similarly, 2 of the 4 PD participants who reported having attempted suicide had never previously vocalized their intent.

#### Associations between prodromal symptoms and STBs

Among the CHR children, bivariate correlations between SBQ-R total score and individual items on the SIPS revealed significant positive correlations between STBs and *Social Anhedonia* (*r* = .47, *p* = .033), *Avolition* (*r* = .48, *p* = .027), *Occupational Functioning* (*r* = .48, *p* = .026), *Odd Behavior or Appearance* (*r* = .57, *p* = .008), *Dysphoric Mood* (*r* = .61, *p* = .003), and *Impaired Tolerance to Normal Stress* (*r* = .55, *p* = .009). When controlling for *Dysphoric Mood* (an item for which the presence of suicidal ideation impacts the score) using partial correlations, only *Social Anhedonia* (*r* = .50, *p* = .024) and *Odd Behavior or Appearance* (*r* = .68, *p* = .001) remained significantly correlated with SBQ-R total score.

#### Discussion

As an exploratory investigation, this study examined suicidal thoughts and behaviors in a subset of youth with psychosis and at clinical high risk for psychosis. To our knowledge, there are no studies that have evaluated STBs in CHR (mean age = 10.6) or psychotic children (mean age = 10.1) of this age. In this preliminary analysis, 10 of 21 CHR children and 11 of 16 PD children reported past-year suicidal ideation. Of concern, 8 of the 37 total participants reported a suicide attempt at one point in their lifetime. These findings provide preliminary evidence that STBs could be as prevalent in CHR and PD children as they are in adolescents [3, 19].

Importantly, these data also suggest that children presenting with psychotic symptoms may underreport the presence of STBs, as evidenced by the 7 children who endorsed either having a suicide plan (n = 5) or having attempted suicide (n = 2) without previously expressing their intent. It is unclear why these children may feel less inclined to talk about their STBs. Nevertheless, these findings suggest that children presenting with psychotic symptoms might be at significant risk for suicide. Careful screening for the presence of STBs, both past and present, should be part of a diagnostic assessment for these youth populations. Clinicians working with CHR and PD youth should also target reduction of STBs in their treatment plans. Cognitive behavioral interventions, which have been shown to be effective in older CHR [20] and PD [21] populations, may need to be adapted in developmentally appropriate ways for younger patients.

While preliminary, when controlling for dysphoric mood, *Social Anhedonia* and *Odd Behavior or Appearance* were the two prodromal symptom categories significantly associated with STB severity in the CHR sample. The SIPS defines *Social Anhedonia* as a lack of close friends or confidants, a preference to spend time alone, or participation in social activities in a disinterested or mechanical way. Of note, a substantial body of literature identifies social anhedonia and/or social isolation as a robust predictor of suicide risk, both in psychotic [22, 23] and non-psychotic populations [24]. That said, it is less clear why *Odd Behavior and Appearance* was associated with STB severity relative to other SIPS items more commonly associated with suicide risk (e.g., *Avolition*, *Impaired Tolerance to Normal Stress*). Future research should further investigate odd behavior or appearance and its contribution to suicide risk among children presenting with psychotic symptoms. No positive prodromal symptoms were significantly correlated with STBs, suggesting that negative and depressive symptoms, more so than positive symptoms, may be associated with suicide risk among CHR populations [5].

## Limitations

This study was exploratory in nature and focused on childhood disorders of lower prevalence. Interpretation of these findings, therefore, is constrained by the small sample size, which resulted in wide confidence intervals and precluded the ability to statistically control for comorbid psychiatric diagnoses. Second, it is possible that comorbid psychiatric diagnoses contributed to the high rates of elevated clinical severity of STBs. In this sample, 4 of the 5 CHR children with SBQ-R scores above the clinical cut score also had a comorbid mood disorder; this was true for 2 of the 6 PD children. Future studies with larger samples should seek to employ more statistical control over potential covariates and utilize longitudinal designs in order to more clearly investigate the relationship between psychotic symptoms and STBs.

## Abbreviations

CHR: clinical high risk
EOP: early onset psychosis
VEOP: very early onset psychosis
PD: psychotic disorder
STBs: suicidal thoughts and behaviors
KSADS-PL: schedule for affective disorders and schizophrenia for school-age children—present and lifetime version
SIPS: structured interview for prodromal syndromes
SBQ-R: Suicide Behaviors Questionnaire—Revised
NOS: not otherwise specified
